# Genome sequencing reveals diversification of virulence factor content and possible host adaptation in distinct subpopulations of *Salmonella enterica*

**DOI:** 10.1186/1471-2164-12-425

**Published:** 2011-08-22

**Authors:** Henk C den Bakker, Andrea I Moreno Switt, Gregory Govoni, Craig A Cummings, Matthew L Ranieri, Lovorka Degoricija, Karin Hoelzer, Lorraine D Rodriguez-Rivera, Stephanie Brown, Elena Bolchacova, Manohar R Furtado, Martin Wiedmann

**Affiliations:** 1 Department of Food Science, Cornell University, Ithaca NY, 14853, USA; 2 Life Technologies Corporation, 850 Lincoln Centre Drive, Foster City CA, 94404, USA; 3 AvidBiotics Corp., 300 Utah Ave., Suite 150, South San Francisco, CA, 94080, USA

## Abstract

**Background:**

Divergence of bacterial populations into distinct subpopulations is often the result of ecological isolation. While some studies have suggested the existence of *Salmonella enterica *subsp. *enterica *subclades, evidence for these subdivisions has been ambiguous. Here we used a comparative genomics approach to define the population structure of *Salmonella enterica *subsp. *enterica*, and identify clade-specific genes that may be the result of ecological specialization.

**Results:**

Multi-locus sequence analysis (MLSA) and single nucleotide polymorphisms (SNPs) data for 16 newly sequenced and 30 publicly available genomes showed an unambiguous subdivision of *S. enterica *subsp. *enterica *into at least two subpopulations, which we refer to as clade A and clade B. Clade B strains contain several clade-specific genes or operons, including a β-glucuronidase operon, a S-fimbrial operon, and cell surface related genes, which strongly suggests niche specialization of this subpopulation. An additional set of 123 isolates was assigned to clades A and B by using qPCR assays targeting subpopulation-specific SNPs and genes of interest. Among 98 serovars examined, approximately 20% belonged to clade B. All clade B isolates contained two pathogenicity related genomic islands, SPI-18 and a cytolethal distending toxin islet; a combination of these two islands was previously thought to be exclusive to serovars Typhi and Paratyphi A. Presence of β-glucuronidase in clade B isolates specifically suggests an adaptation of this clade to the vertebrate gastrointestinal environment.

**Conclusions:**

*S. enterica *subsp. *enterica *consists of at least two subpopulations that differ specifically in genes involved in host and tissue tropism, utilization of host specific carbon and nitrogen sources and are therefore likely to differ in ecology and transmission characteristics.

## Background

*Salmonella *is a human and animal pathogen that causes considerable disease burden worldwide. The genus contains two species, *Salmonella bongori *and *Salmonella enterica*. *S. enterica *can be further subdivided into six subspecies. Of the six subspecies, *Salmonella enterica *subspecies *enterica *is most commonly associated with disease and has undergone the most diversification, as this subspecies comprises approximately 60% of all currently described > 2600 *Salmonella *serovars [1]. In the United States an estimated 11% of the foodborne illnesses are caused by *Salmonella*, which makes it the most prevalent non-viral foodborne pathogen [2]. The two most common disease manifestations of human *Salmonella *infections are gastroenteritis and typhoid fever. Many *Salmonella *serovars can cause a self-limiting gastroenteritis, yet a limited number of *Salmonella *serovars (so-called typhoid serovars), most commonly *S*. Typhi and *S*. Paratyphi A, can cause typhoid fever, a more severe systemic disease [3].

Because of the severity and often fatal consequences of typhoid fever, these serovars have been the subject of comparative genomic and population genetic studies, which indicate that *S*. Typhi and *S*. Paratyphi A are clonal pathogens, and that *S*. Typhi emerged only recently (between 10,000 and 43,000 years ago) [4]. These serovars are only very closely related over 20% of their genome, which is indicative of convergent evolution [5]. They share several pathogenicity islands including SPI-18, a genomic islet encoding the invasion associated TaiA protein and the intracellularly expressed pore-forming hemolysin HlyE [6], and a genomic islet encoding a tripartite cytolethal distending toxin [7].

While serotyping according to the White-Kauffmann-Le Minor scheme (formerly the Kauffman-White scheme [1]) and pulsed field gel electrophoresis are still the subtyping methods of choice in outbreak investigations [8], several approaches have been applied over the last two decades to elucidate the population structure of *S. enterica *subsp. *enterica*, such as multilocus enzyme electrophoresis [9], microarray based gene content assessment [10], amplified fragment length polymorphism [11] and multilocus sequence analysis [12,13]. These studies have greatly improved our understanding of the evolution of *Salmonella*, and, in particular, have shown that while the majority of serovars represent single evolutionary lineages, some serovars (such as Saintpaul [9] Newport, and Kentucky [14,15]), actually occur in several unrelated phylogenetic lineages. None of these studies however demonstrated an unambiguous subdivision of subsp. *enterica *into distinct lineages; for instance Falush *et al. *[13] found two main lineages (clade A and B) in *S. enterica *subsp. *enterica*, however doubted the biological significance of these lineages due to low bootstrap support and extensive allele sharing. A recent study by Parsons *et al. *[16] showed that the subdivision found by Falush *et al. *has biological significance, as the two lineages seem to differ in β-glucuronidase activity, the presence of virulence associated genes and host specificity.

In this report we used a comparative population genomic approach to (i) assess population divergence within *S. enterica *subsp. *enterica*, and (ii) search for differences in gene content putatively associated with ecological divergence of these subpopulations. Additionally we used genomic and PCR-based approaches to (i) map the distribution of SPI-18 and the cytolethal distending toxin islet (called the CdtB-islet from here on) within the *S. enterica *subsp. *enterica *population, and (ii) to investigate the evolutionary relationships between SPI-18 and the CdtB-islet found in non-typoid serovars and typhoid serovars Typhi and Paratyphi A.

## Results and Discussion

### *S. enterica *subsp. *enterica *consists of at least two divergent subpopulations

**Figure 1:**
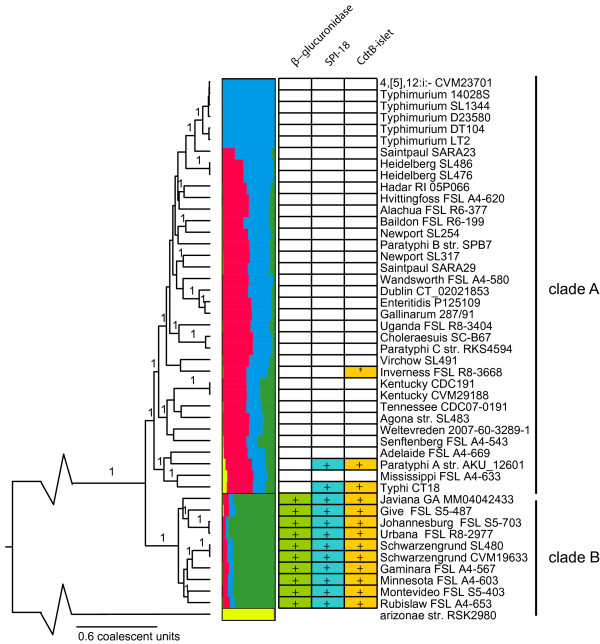
**Phylogenetic relationships of 46 *Salmonella enterica *subsp. *enterica *strains and one *S. enterica *subsp. *arizonae *isolate as inferred by ClonalFrame based on sequence data for 93 loci**. Values above the branches are posterior probabilities and are only indicated when > 0.9. Horizontal bars to the right of the tree indicate mixture of ancestry as inferred by STRUCTURE, the coloration being proportional to the amount of genetic material inherited from a putative ancestral population (red and blue = clade A, green = clade B, yellow = a putative non-subspecies *enterica *ancestor, in this case represented by *S. enterica *subsp. *arizonae*). The blocks to the right of the mixture of ancestry indicate the distribution of the β- glucuronidase operon (green), SPI-18 (blue) and the CdtB-islet (gold) in the genomes. The CdtB-islet in *S*. Inverness has been marked with an asterisk because of its position on a mobile element.

Phylogenetic analysis of 93 randomly selected core genome loci (see Methods) in 16 newly sequenced and 30 publicly available *S. enterica *subsp. *enterica *genomes (additional file 1) as well as one publicly available *S. enterica *subsp. *arizonae *genome revealed two divergent subpopulations of subsp. *enterica*, called clade A and clade B hereafter (Figure 1). The division into two distinct clades is also well supported by a maximum likelihood phylogeny, and a split decomposition phylogenetic network based on all core SNPs in the pan genome (additional file 2), which indicates that phylogenetic signal that supports subdivision of *S. enterica *subsp. *enterica *is not limited to the 93 randomly selected loci, but instead is present genome-wide. Clade B seems to largely overlap with division F identified in a previous multi locus enzyme electrophoresis (MLEE) study [9]. While the subdivision of *S. enterica *subsp. *enterica *into two clades was previously suggested by Falush *et al. *[13], using a traditional seven gene MLST, this subdivision did not achieve strong bootstrap support in this previous analysis due to extensive allele sharing. While this paper was under review, Didelot *et al. *[17] published a population genetic study of *S. enterica *subsp. *enterica *using approximately 300 kbp of sequence data of the core genome for 114 isolates. They identified five well-supported lineages in subsp. *enterica*, of which one (lineage 3) coincides with clade B presented here, while the other four lineages appear as subclades in clade A. Our study, as well as the Didelot *et al. *[17] study, shows that extensive sequence data sets (such as full genome sequence data) are needed to improve our understanding of the evolution of *S. enterica*.

Clade A consists of three highly supported (posterior probability > 0.99) subclades; (i) a subclade consisting of serovars Typhi, Paratyphi A, Mississippi and Adelaide (the Typhi clade), (ii) a subclade consisting of serovars Kentucky, Tennessee, Senftenberg, Weltevreden and Agona (the Agona clade), and (iii) a subclade containing the remainder of the clade A serovars (see Figure 1 and additional file 2). The fact that *S*. Typhi and *S*. Paratyphi A are found in a well supported subclade, suggests that similarity in gene content between these serovars can not only be attributed to convergent evolution due to adaptation to the human host, as suggested by Didelot *et al. *[5], but also reflects shared recent common ancestry.

Although the split of clade B from within *S. enterica *subsp. *enterica *is well supported by a high posterior probabilities (1.0) and high bootstrap values (Figure 1; additional file 2), ancestry analysis [18] of the 93 core genome loci (Figure 1) suggests that allele sharing through horizontal gene transfer and homologous recombination between the clades has occurred, but not to the extent that divergence between the two clades has been eliminated. A similar pattern of divergent lineages in the face of high recombination rates has been observed in *Escherichia coli *[19], which suggests that this may be a common pattern in enteric bacteria related to host niche specialization. Isolates representing some clade A serovars (i.e., Senftenberg, Kentucky, Agona, Tennessee, Weltevreden) possess a high proportion (between 21 and 32%) genetic material from clade B, suggesting that they represent intermediate genotypes, an interpretation that is also supported by SNP-based phylogenies (additional file 2). As our population genetic analysis suggested that most members of the Typhi clade (except Adelaide) may share a considerable amount of genetic material (between 5 and 9%) with *S. enterica *subsp. *arizonae*, pairwise comparisons between *S*. Typhi and *S. enterica *subsp. *arizonae *genomes were performed. Among 2,241 shared ORFs, only 23 showed pairwise nucleotide divergence < 1%, providing no evidence for a significant proportion of highly conserved genes. This suggests that members of the Typhi clade received genetic material from *S. enterica *subspecies other than subsp. *arizonae*. Genome sequencing of additional *S. enterica *subspecies will be necessary to test this hypothesis.

### Clade-specific genes suggest differences in adhesion, colonization properties, and metabolic capabilities between *S. enterica *subsp. *enterica *clades

**Figure 2:**
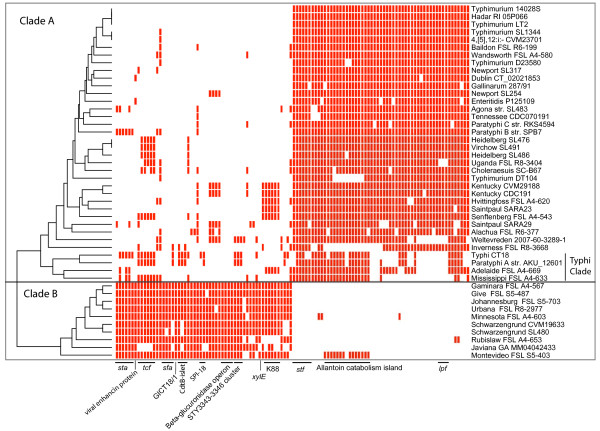
**Heatmap of gene presence (red) and absence (white) of genes enriched in their presence in clade A or clade B**. The cluster diagram on the left is based on the presence/absence data of the enriched genes. Genes were ordered according to their position in the reference genomes. Fimbrial operons are indicated with their standard locus names; *sta*, *tcf*, *sfa *and K88 for clade B, and *stf *and *lpf *for clade A.

Genes that are differentially maintained in bacterial subpopulations are likely to reveal differential selective pressures acting on these subpopulations [20]. To identify putative differentially maintained genes we searched for genes that were either clade A or clade B enriched (see Methods section and Figure 2). The most abundant group of genes that were enriched in either clade were genes associated with fimbrial operons. We found two fimbrial operons (*stf *and *lpf*) to be conserved among clade A genomes, and absent among clade B genomes, while we found one clade B specific fimbrial operon (*sfa*), and three fimbrial operons (*sta*, *tcf*, and a K88-like fimbrial operon) that were clade B enriched. This differential distribution of fimbrial operons suggests that the two clades differ in their adhesion abilities.

Another gene indicative of putative niche differentiation between the two clades encodes a putative metalloprotease with homology to Bacterial and Baculoviridae enhancins; this gene is present in all clade B genomes, but found in only 2/36 clade A genomes. This protein not only increases infectivity of Baculoviridae by degrading the peritrophic membrane of insect midguts [21], but a homolog of this enhancin has also been found in the genome of *Yersinia pestis *[22], which survives in both mammals and insect host. The presence of this gene therefore suggests that insects may play a role as alternate hosts for clade B strains.

**Figure 3:**
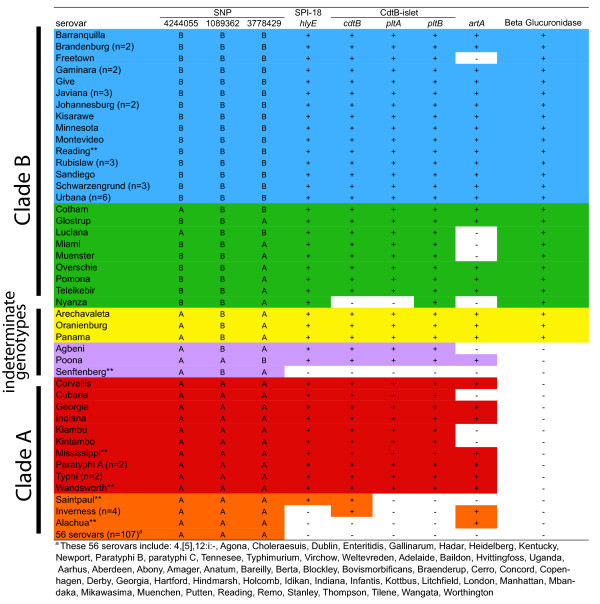
**Distribution of clade specific SNPs, Typhi-associated virulence genes and β- glucuronidase activity among 169 *S. enterica *subsp. *enterica *isolates representing 98 serovars**. Clade specific SNPs and the presence of Typhi associated virulence genes were determined using SNP assays, TaqMan^® ^assays or full genome sequence data. β- glucuronidase activity was determined using a phenotypic assay. If multiple isolates representing the same serovar had the same characteristics the number of isolates is indicated by n = in parenthesis, ** indicates that multiple isolates of the same serovar had different characteristics. Serovars colored blue have three clade B specific SNPs, light green indicates serovars with two out of three clade B specific SNPs, yellow indicates serovars which are β-glucuronidase positive and have one out of three clade B specific SNPs, purple indicates serovars which are β-glucuronidase negative and have one out of three clade B specific SNPs, red indicates serovars that have three clade A specific SNPs and tested positive for SPI-18 and the CdtB-islet, and orange indicates serovars that have three clade A specific SNPs and that tested positive for one or two toxin related genes.

We further found differences among clade A and clade B in the presence of two genomic regions involved in nitrogen and carbon metabolism, the β-glucuronidase operon in clade B and the allantoin catabolism island in clade A. The β-glucuronidase operon is exclusively found in clade B genomes and enables the organism to use glucuronides as an additional carbon source. Because glucuronides are typically found in the gall bladder [23] and gut environment of vertebrate hosts [24,25], β-glucuronidase-positive strains may have a fitness advantage in the vertebrate gastrointestinal environment. To prove this fitness advantage, additional competition experiments involving the comparison of the fitness of wild type strains and β-glucuronidase deletion mutants in a vertebrate host are necessary. Phenotypic screening for β-glucuronidase activity using a fluorogenic substrate indicated that this locus is functional in all clade B strains (Figure 3).

The allantoin catabolism island confers the ability to use allantoin as the sole carbon and nitrogen source [26], and is found to be present, in its entirety, in 73% of the clade A genomes, and absent in clade B, with the exception of the genome of the Montevideo strain we sequenced. This is putatively an adaptation to the mammalian host, as allantoin is the end-product of the purine catabolic pathway of most mammals, with the exception of Hominoids [27]. In Hominoids, birds and most reptiles the end-product of the purine catabolic pathway is uric acid. As pointed out by Kingsley *et al. *[28], the allantoin catabolism island is non-functional due to gene degradation in some genomes of human (Typhi and Paratyphi A) and bird (Gallinarum) restricted serovars, possibly due to it being obsolete for survival in non-allantoin producing hosts. Conversely, the allantoin catabolism cluster may be conserved in genomes of serovars that are adapted to allantoin-producing hosts, such as *S*. Dublin, a serovar adapted to bovine hosts [29]. Gene degradation is observed in the allantoin catabolism cluster in two strains of *S*. Typhimurium (D23580 [28] and DT104) and may therefore indicate that these strains may have shifted from a broad host range associated with *S*. Typhimurium [29] or cattle adapted [30] as in *S*. Typhimurium DT104, to being more (human) host specific. However, this correlation of gene degradation in the allantoin catabolism cluster and host specificity does not seem to be a general rule. The genomes of human host specific serovars such as Paratyphi B and Paratyphi C have a full allantoin catabolism cluster, while gene degradation within the cluster is observed in *S*. Cholerasuis, a serovar predominantly associated with pigs [29], which are allantoin producing hosts [31].

### Several typhoid-associated pathogenicity islands are also conserved in clade B serovars

*Salmonella *pathogenicity islands (SPIs) and islets are horizontally acquired genomic islands that encode virulence factors and other proteins important for pathogenesis, persistence in the host, and host specificity [32]. The distribution of SPIs may be indicative of potential differences in niche specialization among different *Salmonella *strains. In total, eight SPIs/islets (SPI-1, SPI-2, SPI-4, SPI-5, SPI-9, SPI-11, SPI-12 and CS54) were found to be present in all sequenced serovars. On the opposite end of the spectrum, four SPIs were restricted to a single sequenced serovar; such as SPI-7 and SPI-15 (only found in *S*. Typhi), and SPIs 20-21 (only found in *S. enterica *subsp. *arizonae*). Another four SPIs (SPI-8, 10, 17 and 19) were found to have limited presence and were identified in only two to eight (< 18%) of sequenced serovars. Interestingly, among these 8 serovar restricted and limited SPIs, five (SPI-7, 8, 10, 15, and 17) were associated with human-specific typhoid causing serovars, i.e., *S*. Paratyphi A and/or *S*. Typhi, while two (20, and 21) were associated with *S. enterica *subsp. *arizonae*. The remaining 8 SPIs/islets were observed to have a mixed pattern of distribution, being present in at least six serovars.

Further analysis of the 8 SPIs with mixed distribution patterns identified three pathogenicity islands (SPI-18, the CdtB-islet, and GICT18/1) that were present in all clade B isolates as well as *S*. Typhi, and *S*. Paratyphi A, but absent from all other clade A isolates. SPI-18 encodes two genes important for pathogenesis including *hlyE*, encoding an intracellularly expressed pore-forming hemolysin, and *taiA*, encoding an invasion associated protein [6]. The CdtB-islet or typhoid toxin islet that was previously described by Spano *et al. *[7], encodes a tripartite toxin consisting of a cytolethal distending toxin subunit B (CdtB) and two pertussis-like toxin subunits (PltA and PltB). In *S*. Typhi, *S*. Paratyphi A and clade B strains the CdtB-islet is found in SPI-11. A divergent CdtB-islet was also found in *S*. Inverness (where it is on a putative mobile element) and *S. enterica *subsp. *arizonae*; in both of these strains the CdtB-islet was in a different chromosomal location as compared to the clade B strains and *S*. Typhi and *S*. Paratyphi A. A search in the SEED-viewer (http://seed-viewer.theseed.org) also revealed a divergent CdtB-islet in *S. bongori *(GenBank accession number FR877557; SBG_1077 - SBG_1081). In almost all examined strains the GICT18/1 islet is inserted in the *sapABCDF *operon; this insertion has previously been shown to disrupt antimicrobial peptide resistance [33]. GICT18/1 harbors another two-subunit pertussis-like toxin, encoded by *artA *and *artB *[33], which have 59 and 73% nucleotide sequence identity, respectively, to *pltA *and *pltB *in the CdtB-islet [34]. *S*. Inverness carries the *artAB *locus, but on a putative prophage, much like the *artAB *islet previously reported in *S*. Typhimurium DT104 [34].

In addition to the virulence islands described above, we also found a cluster of four putative membrane and exported proteins (*S*. Typhi genes STY3343 - STY3346) that was present in all clade B serovars as well as *S*. Typhi, *S*. Paratyphi A and *S*. Weltevreden, but absent from other clade A serovars. The functional relevance of this gene cluster is unknown, but the fact that its distribution mirrors that of SPI-18, the CdtB-islet, and to a large extent GICT18/1 suggests that these genes may also play a role in the virulence of clade B, *S*. Typhi, and *S*. Paratyphi A.

### Distribution of clade B isolates and typhoid-associated pathogenicity islands among *S. enterica *subsp. *enterica serovars*

To further investigate the distribution, among the S. *enterica *subsp. *enterica *population, of SPIs and islets shared between clade B, *S*. Typhi, and S. Paratyphi A, targeted screening was performed against a panel of 123 isolates, expanding the number total number of characterized serovars to 98. Clade membership was assessed using a set of three genotyping assays targeting clade B specific SNPs, while TaqMan assays targeting *hlyE *(SPI-18), *cdtB, pltA, and pltB *(the CtdB-islet), and *artA *(GICT18/1) were used to screen for the presence of three islands (Methods). In addition, strains were screened for β-glucuronidase activity, a clade B-specific phenotype linked to the presence of the β-glucuronidase operon (see above). Isolates representing 24 serovars (including the nine already classified into clade B based on full genome sequence data; see Figure 1) had two or three clade B specific SNP alleles and were therefore considered to be members of clade B (Figure 3). The *hlyE, cdtB, pltA*, and *pltB *loci were present in 95% of isolates classified into clade B, the only exception being a *S*. Nyanza isolate, which did not yield a *cdtB *or *pltA *PCR amplicon and may thus harbor an incomplete CtdB-islet. While *artA *was not detected in five clade B isolates (Figure 3), all of these isolates were positive for β-glucuronidase activity.

Isolates representing 41 serovars (in addition to the 28 already classified in Figure 1) had three clade A SNP alleles and were β-glucuronidase negative, indicating putative membership in clade A. *artA *was found in a few isolates classified as clade A; the irregular presence of the *artAB *toxin locus in both clades A and B may be due to the fact that this locus can be found on prophages [34]. While the majority of clade A isolates (108/123) did not encode SPI-18 and the CtdB-islet, 10 of the clade A isolates in our panel were positive for both of these pathogenicity islets (Figure 3). These isolates included, as expected, serovars Paratyphi A and Typhi, but surprisingly also included serovars Mississippi and Wandsworth, two serovars for which the sequenced isolates did not harbor SPI-18 or the CtdB-islet. Independent MLST data (obtained from http://mlst.ucc.ie/mlst/dbs/Senterica) for three *S*. Mississippi isolates are consistent with the existence of two clonal complexes, with one isolate differing from the other two at six of the seven loci; we thus propose that the Mississippi strains in the screening panel and the sequenced strain may represent two distinct clonal groups. As only a single *S*. Wandsworth isolate is present in the MLST database, we could not further confirm that this serovar is also polyphyletic. Additional clade A serovars that harbor SPI-18 and the CdtB-islet are Corvallis, Cubana, Georgia, Indiana, Kiambu, and Kintambo (Figure 3). As sequence information is sparse or non-existent for these serovars, determining their phylogenetic relationship to *S*. Typhi and *S*. Paratyphi A will require additional sequence data. None of these clade A SPI-18 and CdtB-islet positive serovars has been linked to a large number of disease cases; among these, the serovar most frequently associated with human disease cases in the US (based on 1996 to 2006 data) was Kiambu with a total of 409 cases (of 390,767 total cases) (http://www.cdc.gov/ncidod/dbmd/phlisdata/salmonella.htm). While this suggests that the presence of SPI-18 and CdtB-islet in a clade A strain is not necessarily associated with increased virulence, we did observe a striking correlation between the presence of SPI-18 and the presence of CdtB-islet across clades A and B (Figure 3). All 54 isolates (representing 38 serovars; see Figure 3) that carried a full CdtB-islet (as indicated by positive PCR assays for *cdtB*, *pltA *and *pltB*) also have the SPI-18 gene *hlyE*; only two of the 56 strains with the *hlyE *locus lack a full CdtB-islet. Future experimental work will be needed to clarify if these two loci may be functionally co-dependent, particularly in non-typhoid serovars.

Isolates representing 6 serovars had one clade B and two clade A SNP alleles, indicating ambiguous clade membership. One of these isolates, a *S*. Senftenberg strain unrelated to the sequenced strain, did not harbor SPI-18 (*hlyE*) or the CdtB-islet (*cdtB, pltA *and *pltB*). Three isolates representing *S*. Panama, *S*. Oranienburg and *S*. Arechavaleta were positive for SPI-18, the CdtB-islet and exhibited β-glucuronidase activity, while *S*. Agbeni and *S*. Poona were positive for SPI-18 and the CdtB-islet, but did not exhibit β-glucuronidase activity (Figure 3). This supports that a small proportion of the *S. enterica *subsp. *enterica *population may be of mixed clade A and clade B origin.

Serovar Reading included two isolates, one each classified into clade B (three clade B SNPs, SPI-18, CdtB-islet, and β-glucuronidase positive) and clade A (three clade A SNPs, SPI-18, CdtB-islet, and β-glucuronidase negative). We propose that this serovar may be polyphyletic, as has been previously observed for *S*. Saintpaul and *S*. Newport [15], further indicating that isolates with identical serovars may not always represent the same clonal group.

We found that among 98 serovars examined, approximately 25% belonged to clade B. This may be explained by the fact that we examined isolates that were almost entirely derived from human clinical cases. Parson *et al. *[16] found that clade A was the prevalent clade among isolates from mammals (67% of the isolates), while clade B was significantly overrepresented (80%) among isolates from reptiles. The high prevalence of clade A isolates in our study is most likely an effect of differences in host specificity of clade A and clade B.

### Typhi virulence gene repertoire consists of a unique combination of ancestral and newly acquired genes

Horizontal gene transfer is known to have contributed to the evolution of *S*. Typhi and *S*. Paratyphi A [5]. Over 20% of the genome of these two serovars is nearly identical, indicating recent, extensive gene sharing [5]. Furthermore, the Vi capsular operon of *S*. Typhi is 99.88% identical to that of *S*. Paratyphi C, which is only distantly related, though the direction of this putative transfer is unknown [35]. The presence of SPI-18, the CdtB-islet, and GICT18/1 in only a small subset of clade A strains, including *S*. Typhi and *S*. Paratyphi A, suggests that these loci may have been horizontally transferred from a clade B strain into those strains. Evidence for recombination breakpoints was found in all three islands and therefore the phylogeny for each non-recombinant block within the islands was analyzed independently. The SPI-18 phylogeny, omitting the first 494 nt, which are upstream of *hlyE *and *taiA*, shows a deep split between clade B and the Typhi and Paratyphi A clade (additional file 3). A similar topology is observed for the *artAB *toxins in GICT18/1, and most of the CdtB-islet, with the exception of *pltB *(see below). The deep branching between clade B and the Typhi and Paratyphi A clade indicates that these islands have not been transferred between the clades, at least not subsequent to the diversification of clade B.

In order to probe for evidence for large scale horizontal gene transfer between clade B strains and *S*. Typhi, pairwise comparisons of gene similarities between nine clade B genomes and *S*. Typhi were computed, and nucleotide divergence histograms were plotted (additional file 4). Didelot, *et al. *[5] previously used a similar approach to probe for large scale horizontal gene transfer in *S*. Typhi and Paratyphi A, but at the time, no clade B genomes were available for comparison. Our analyses did not reveal a class of genes that were highly conserved between clade B genomes and Typhi (additional file 4), indicating that extensive recent allele sharing has not occurred between any of these clade B strains and *S*. Typhi. As further evidence against recent horizontal transfer of the SPI-18, the CdtB-islet, and GICT18/1 genomic islands, these loci are more divergent between clades than the average gene. For example, nucleotide divergence values of SPI-18, the CdtB-islet, and GICT18/1 between *S*. Schwarzengrund (clade B) and *S*. Typhi are 3.4%, 3.0%, and 2.6%, respectively, all much higher than the genome-wide average divergence of 1.3%. None of these islands are among the 20% of the genome that has been recently exchanged between *S*. Typhi and *S*. Paratyphi A [5]. Based on these results, it is likely that these islands have been vertically inherited from the most recent common ancestor (MRCA) of clades A and B. However, horizontal transfer from an ancient clade B ancestor to the *S*. Typhi clade cannot be ruled out. Though most of the sequence in these islands appears not to have been horizontally transferred, a 488 nt fragment of GICT18/1 encoding an integrase, and a 728 nt fragment of the *pltB *gene in the CdtB-islet have phylogenies consistent with horizontal transfer from a clade B strain to *S*. Paratyphi A (additional file 3). Overall, our data thus suggests that the unusual virulence phenotype of *S*. Typhi and *S*. Paratyphi A can be attributed to a unique combination of recently acquired virulence factors, such as the mobile Vi capsular operon in *S*. Typhi, and ancestral conserved virulence factors such as SPI-18 and the CdtB-islet.

## Conclusions

In this study we provide further evidence that *Salmonella enterica *subsp. *enterica *represents two evolutionary clades with distinct genome characteristics, including different virulence gene repertoires, suggesting that transmission characteristics and ecology are distinct between these groups. This study will provide a population genetic framework for future studies on the evolution of virulence and transmission characteristics in *Salmonella enterica*. As the commonly studied non-typhoidal *Salmonella *serovars (i.e., Enteritidis, Typhimurium, Newport), all represent clade A strains, our study specifically suggests a need to include a clade B strain in future studies of *Salmonella *pathogenesis and virulence.

## Methods

### Bacterial isolates

A total of 16 isolates representing different *Salmonella *serovars (i.e., Montevideo, Inverness, Rubislaw, Give, Mississippi, Urbana, Uganda, Senftenberg, Gaminara, Baildon, Minnesota, Hvittingfoss, Adelaide, Alachua, Wandsworth, and Johannesburg; additional file 1) were selected for full genome sequencing.

### DNA sequencing and assembly

DNA sequencing and de novo assembly was performed using the Applied Biosystems SOLiD™ system as previously described [36], using the complete genomes of *S*. Enteritidis P125109 (GenBank accession NC_011294) and *S*. Typhimurium 14028S (CP001363) as reference for scaffolding of the de novo assembled contigs. Pseudogenomes were prepared and submitted to RAST [37] for automatic annotations (http://rast.nmpdr.org/). After automatic annotation of the genomes, the annotations were manually curated with an emphasis on mobile elements and pathogenicity related genes. GenBank accession numbers are provided in additional file 1; assembly statistics are given in additional file 5. ORFs from each strain were compared to the GenBank non-redundant protein database by BLASTX, and when two adjacent ORFs, separated by a sequence gap, were found to match adjacent fragments of the same subject protein, the ORF sequences were merged, in frame, for downstream analyses. The SOLiD™ system is intended for research use only and is not intended for animal or human therapeutic or diagnostic use.

### Genome comparison, identification of unique genomic regions and clade enriched genes

Sequences unique to groups of genomes were identified using the MUMmer software package [38] to compare inclusion and exclusion genomes to a single reference sequence. A custom Perl script identified sequences of at least 100 nt that are at least 95% identical in all inclusion strains, and that do not match any exclusion strains with greater than 80% identity. Orthologous genes in the genomes were found using a all-against-all BLAST approach as implemented in R [39] scripts of L. Snipen [40]. The pangenome matrix resulting from these scripts was used for the enrichment analysis. A clade gene enrichment statistic was computed for each group of orthologous genes. This statistic is as follows: Cs = (n_A_/A)-(n_B_/B), where Cs is the clade gene enrichment statistic, n_A _is the number of clade A genomes in which a certain ortholog is present, A is the total number of clade A genomes, n_B _is the number of clade B genomes in which this ortholog is present and B is the total number of clade B genomes. This statistic is -1 when a certain ortholog is only present in clade B and 1 when a certain ortholog is only present in clade A. The cutoff value of Cs was chosen to obtain the lowest 0.5% and the highest 0.5%. These orthologs were manually annotated and checked for putative artifacts resulting from the use of draft genomes. An overview of the ORFs found in this analysis can be found in additional file 6.

### Phylogenetic and population genetic analyses

For the construction of an intraspecific phylogeny, 100 loci were initially selected from a randomized list of genes found in the core genome of 30 *S. enterica *subspecies *enterica *strains and one subsp. *arizonae *strain. Genes that were previously shown be under positive selection and/or recombination [41], or were part of a multicopy gene family or highly fragmented due to assembly artifacts were excluded from this selection. Seven loci were excluded from the analyses because they were not present in some of the 16 draft genomes that were added to the analysis in a later stage of the study. Initial sequence alignments of the individual genes were created in RAST using CLUSTAL W version 1.8.3 [42]. These alignments were all individually inspected and trimmed to match the length of the shortest ORF in the alignment. Large insertion deletion regions were excluded from the alignments. The XMFA formatted file of the alignments can be found in additional file 7. A phylogeny based on these 93 loci (representing 73,761 nt in total) was inferred using Clonalframe version 1.2 [43]; six individual runs of 50,000 generations pre-burn-in and 250,000 generations post-burn-in were performed with the scaled mutational rate θ set equal to Watterson's moment estimator and the mean tract length of imported sequence fragments fixed to 1,268 nt for all runs. Two runs were discarded because of improper convergence, and the trees of the four properly converged runs (as determined by the Gelman-Rubin test [44]) were combined to produce a summary tree using TreeAnnotator (part of the BEAST package [45]). Mixture of ancestry as inferred from the 93 loci data set by STRUCTURE version 2.3.3 [46], using the linkage model [18]. Initially five individual runs with a K value of 2 to 6, and with 10,000 pre-burn-in and 20,000 post burn-in generations were performed to find the optimal K- value. The K-value for which the highest likelihood of observing data was found (K = 4) was used for three runs of 50,000 pre-burn-in and 100,000 post burn-in generations. The results of the run with the highest likelihood score of observing the data are shown in Figure 1.

SNPs were identified using the MUMmer software package [38] and core SNPs (those in chromosomal regions that are present in all *S. enterica *subsp. *enterica *genomes) were compiled using Perl scripts. Maximum likelihood SNP trees were inferred with RaxML 7.0.4 [47] using the rapid bootstrap procedure to infer non-parametric bootstrap values.

Recombination/breakpoint analyses for SPI-18, the CdtB-islet and GICT18/1 were performed by using GARD [48]. Maximum likelihood phylogenies for the individual fragments between breakpoints were inferred with PAUP* 4.010b [49].

### Quantitative PCR analyses

Primers and probes used for the quantitative PCR based population screen can be found in additional file 8. SNPs were detected using TaqMan^® ^genotyping assays, which have one dye-labeled probe targeting each of the two alleles. Endpoint results of SNP assays were evaluated using TaqMan^® ^Genotyper software (Applied Biosystems), which calls genotypes based on the relative intensity of the two fluorescent dyes.

### Phenotypic analyses

β-glucuronidase activity was determined by growing the isolates in EC MUG broth (EMD Chemicals Inc., Gibbstown NJ) for 16 hours, after which the presence of the fluorogenic product 4-methylumbelliferone was quantified using a Packard fusion instrument (Perkin-Elmer).

## Authors' contributions

AMS, KH, MRF, and MW designed research; HCdB, AMS, GG, CAC, MLR., LD, LDR-R, SB, and EB performed research; HCdB, AMS, GG., and CAC analyzed data; HCdB, GG, CAC, and MW wrote the paper. All authors read and approved the final manuscript.

## Supplementary Material

Additional file 1 **Table of Genomes and strains used in comparative genomic and phylogenomic analysis**. Word document containing list of genomes and strains used in comparative genomic and phylogenomic analysis. Click here for file

Additional file 2 **SNP-based maximum likelihood tree and NeighborNet phylogenetic network inferred from 8,779 SNPs found in the *S. enterica *subsp. *enterica *core genome**. PDF file containing results of phylogenetic analyses of core genome SNPs. Click here for file

Additional file 3 **Phylogenetic trees for individual genes and/or regions, without significant signal for recombination, located in SPI-18, the CdtB-islet and the GICT18/1 (artAB) islet**. PDF file containing results of phylogenetic analyses of individual genes and/or regions, without significant signal for recombination, located in SPI-18, the CdtB-islet and the GICT18/1 (*artAB*) islet. Click here for file

Additional file 4 **Histogram of pairwise nucleotide divergence for selected combinations of *Salmonella *serovars**. PDF file containing histograms of pairwise nucleotide divergence for selected combinations of *Salmonella *serovars. Click here for file

Additional file 5 **Draft genome assembly statistics for 16 *S. enterica *strains newly sequenced in this study**. Word document containing an overview of the assembly statistics of the 16 newly sequenced *Salmonella *genomes. Click here for file

Additional file 6 **Overview genes enriched in either clade B or A of *Salmonella enterica *subsp. *enterica***. Excel file with overview genes enriched in either clade B or A of *Salmonella enterica *subsp. *enterica*. Click here for file

Additional file 7 **Alignments of the 93 loci used in the ClonalFrame analysis**. Text file with alignments of the 93 loci used in the ClonalFrame analysis in XMFA format. Click here for file

Additional file 8 **Primers and probes used for quantitave PCR experiments**. Word document containing sequences of primers and probes used for quantitave PCR based population screen Click here for file
