# Supplementary material for: Genome sequencing reveals diversification of virulence factor content and possible host adaptation in distinct subpopulations of Salmonella enterica
Source: BMC Genomics. 2011 Aug 22;12:425. doi: 10.1186/1471-2164-12-425 (PMC3176500; doi:10.1186/1471-2164-12-425)
Supplement: Additional file 1 — Table of Genomes and strains used in comparative genomic and phylogenomic analysis. Word document containing list of genomes and strains used in comparative genomic and phylogenomic analysis. [file 1471-2164-12-425-S1.DOC]

Additional file 1. Table of Genomes and strains used in comparative genomic and phylogenomic analysis

| isolate/Strain^a^ | Serovar | | Genbank accession^b^ | clade^c^ |
| --- | --- | --- | --- | --- |
| *Publicly available genome sequences:* | | |  |  |
| **subsp. *enterica*** | |  |  |  |
| CVM23701 | | 4,[5],12:i:- | ABAO00000000 | A |
| SL483 | | Agona | CP001138 | A |
| SC-B67 | | Choleraesuis | AE017220 | A |
| CT_02021853 | | Dublin | CP001144 | A |
| P125109 | | Enteritidis | AM933172 | A |
| 287/91 | | Gallinarum | AM933173 | A |
| RI_05P066 | | Hadar | ABFG00000000 | A |
| SL476 | | Heidelberg | CP001120 | A |
| SL486 | | Heidelberg | ABEL00000000 | A |
| GA MM04042433 | | Javiana | ABEH00000000 | B |
| CDC 191 | | Kentucky | ABEI00000000 | A |
| CVM29188 | | Kentucky | ABAK00000000 | A |
| SL254 | | Newport | CP001113 | A |
| SL317 | | Newport | ABEW00000000 | A |
| AKU_12601 | | Paratyphi A | FM200053 | A |
| SPB7 | | Paratyphi B | CP000886 | A |
| RKS4594 | | Paratyphi C | CP000857 | A |
| SARA23 | | Saintpaul | ABAM00000000 | A |
| SARA29 | | Saintpaul | ABAN00000000 | A |
| CVM19633 | | Schwarzengrund | CP001127 | B |
| SL480 | | Schwarzengrund | ABEJ00000000 | B |
| CDC07-0191 | | Tennessee | ACBF00000000 | A |
| CT18 | | Typhi | AL513382 | A |
| 14028S | | Typhimurium | CP001363 | A |
| D23580 | | Typhimurium | FN424405 | A |
| DT104 NCTC 13348 | | Typhimurium |  | A |
| LT2 | | Typhimurium | AE006468 | A |
| SL1344 | | Typhimurium | FQ312003 | A |
| SL491 | | Virchow | ABFH00000000 | A |
| 2007-60-3289-1 | | Weltevreden | ABFF00000000 | A |
| **subsp. *arizonae*** | |  |  |  |
| RSK2980 | | 62:z4,z23:-- | CP000880 | n/a |
|  | |  |  |  |
| *Newly sequenced strains:* | | |  |  |
| **subsp. *enterica*** |  | |  |  |
| FSL A4-669 | Adelaide | | AFCI00000000 | A |
| FSL R6-377 | Alachua | | AFCJ00000000 | A |
| FSL R6-199† | Baildon | | AFCK00000000 | A |
| FSL A4-567 | Gaminara | | AFCL00000000 | B |
| FSL S5-487† | Give | | AFCM00000000 | B |
| FSL A4-620 | Hvittingfoss | | AFCN00000000 | A |
| FSL R8-3668† | Inverness | | AFCO00000000 | A |
| FSL S5-703 | Johannesburg | | AFCP00000000 | B |
| FSL A4-603 | Minnesota | | AFCQ00000000 | B |
| FSL A4-633† | Mississippi | | AFCR00000000 | A |
| FSL S5-403† | Montevideo | | AFCS00000000 | B |
| FSL A4-653† | Rubislaw | | AFCT00000000 | B |
| FSL A4-543 | Senftenberg | | AFCU00000000 | A |
| FSL R8-3404 | Uganda | | AFCV00000000 | A |
| FSL R8-2977 | Urbana | | AFCW00000000 | B |
| FSL A4-580 | Wandsworth | | AFCX00000000 | A |
|  |  | |  |  |
| *Strains used for population wide screens:* | | |  |  |
| **subsp. *enterica*** |  | |  |  |
| FSL R8-0153 | Aarhus | |  | A |
| FSL R8-3524 | Aberdeen | |  | A |
| FSL S5-469 | Abony | |  | A |
| FSL S5-551 | Adelaide | |  | A |
| FSL S5-417 | Agbeni | |  | X |
| FSL S5-517 | Agona | |  | A |
| FSL R8-2924 | Alachua | |  | A |
| FSL A4-650 | Amager | |  | A |
| FSL S5-540 | Anatum | |  | A |
| FSL S5-453 | Arechavaleta | |  | X |
| FSL R8-2449 | Bareilly | |  | A |
| FSL R8-1295 | Barranquilla | |  | B |
| FSL R8-2917 | Berta | |  | A |
| FSL S5-648 | Blockley | |  | A |
| FSL A4-577 | Bovismorbificans | |  | A |
| FSL S5-373 | Braenderup | |  | A |
| FSL R8-1984 | Brandenburg | |  | B |
| FSL R8-4801 | Brandenburg | |  | B |
| FSL R8-0370 | Cerro | |  | A |
| FSL R8-0938 | Cerro | |  | A |
| FSL R8-6332 | Choleraesuis | |  | A |
| FSL R8-0457 | Concord | |  | A |
| FSL S5-786 | Copenhagen | |  | A |
| FSL R8-0092 | Corvallis | |  | A |
| FSL R8-0792 | Cotham | |  | B |
| FSL S5-632 | Cubana | |  | A |
| FSL R8-2630 | Derby | |  | A |
| FSL R6-535 | Dublin | |  | A |
| FSL R6-607 | Dublin | |  | A |
| FSL R8-1271 | Dublin | |  | A |
| FSL R8-1572 | Dublin | |  | A |
| FSL R8-1599 | Dublin | |  | A |
| FSL R8-2962 | Dublin | |  | A |
| FSL R8-3349 | Dublin | |  | A |
| FSL R8-3568 | Dublin | |  | A |
| FSL R8-3570 | Dublin | |  | A |
| FSL R8-4015 | Dublin | |  | A |
| FSL R8-4423 | Dublin | |  | A |
| FSL R8-4810 | Dublin | |  | A |
| FSL S5-407 | Dublin | |  | A |
| FSL S5-439 | Dublin | |  | A |
| FSL A4-670 | Ealing | |  | A |
| FSL S5-415 | Enteritidis | |  | A |
| FSL S5-483 | Enteritidis | |  | A |
| FSL S5-668 | Freetown | |  | B |
| FSL R8-2934 | Gaminara | |  | B |
| FSL R6-992 | Georgia | |  | A |
| FSL R8-2600 | Glostrup | |  | B |
| FSL S5-543 | Hadar | |  | A |
| FSL A4-617 | Hartford | |  | A |
| FSL S5-448 | Heidelberg | |  | A |
| FSL S5-480 | Heidelberg | |  | A |
| FSL R8-3386 | Hindmarsh | |  | A |
| FSL R6-227 | Holcomb | |  | A |
| FSL R8-0789 | Hvittingfoss | |  | A |
| FSL R8-0091 | Idikan | |  | A |
| FSL R6-527 | Indiana | |  | A |
| FSL S5-734 | Infantis | |  | A |
| FSL R8-3669 | Inverness | |  | A |
| FSL R8-3670 | Inverness | |  | A |
| FSL R8-3671 | Inverness | |  | A |
| FSL S5-395 | Javiana | |  | B |
| FSL S5-406 | Javiana | |  | B |
| FSL R8-3499 | Johannesburg | |  | B |
| FSL S5-273 | Kentucky | |  | A |
| FSL R6-203 | Kiambu | |  | A |
| FSL S5-712 | Kintambo | |  | A |
| FSL A4-595 | Kisarawe | |  | B |
| FSL R8-2447 | Kottbus | |  | A |
| FSL R8-2112 | Litchfield | |  | A |
| FSL R8-0459 | London | |  | A |
| FSL R8-3555 | Luciana | |  | B |
| FSL R6-542 | Manhatan | |  | A |
| FSL R8-1550 | Manhatan | |  | A |
| FSL R8-2473 | Manhatan | |  | A |
| FSL R8-2480 | Manhatan | |  | A |
| FSL R8-2498 | Manhatan | |  | A |
| FSL R8-1303 | Manhattan | |  | A |
| FSL S5-451 | Mbandaka | |  | A |
| FSL R8-2520 | Miami | |  | B |
| FSL R6-244 | Mikawasima | |  | A |
| FSL R8-2455 | Mississippi | |  | A |
| FSL S5-504 | Muenchen | |  | A |
| FSL S5-432 | Muenster | |  | B |
| FSL S5-639 | Newport | |  | A |
| FSL S5-654 | Nyanza | |  | B |
| FSL S5-642 | Oranienburg | |  | X |
| FSL R8-0144 | Overschie | |  | B |
| FSL R8-2486 | Panama | |  | X |
| FSL R6-883 | Paratyphi A | |  | A |
| FSL S5-447 | Paratyphi B var. Java | |  | A |
| FSL R6-305 | Paratyphi C | |  | A |
| FSL S5-481 | Pomona | |  | B |
| FSL R8-1546 | Poona | |  | X |
| FSL A4-590 | Putten | |  | A |
| FSL A4-832 | Reading | |  | B |
| FSL R8-1987 | Reading | |  | A |
| FSL R8-3521 | Remo | |  | A |
| FSL R8-4461 | Rubislaw | |  | B |
| FSL S5-477 | Rubislaw | |  | B |
| FSL S5-649 | Saintpaul | |  | A |
| FSL A4-827 | Sandiego | |  | B |
| FSL S5-458 | Schwarzengrund | |  | B |
| FSL S5-658 | Senftenberg | |  | X |
| FSL S5-408 | Stanley | |  | A |
| FSL R8-1526 | Telelkebir | |  | B |
| FSL R8-1965 | Tennessee | |  | A |
| FSL R8-2984 | Thompson | |  | A |
| FSL R8-3268 | Thompson | |  | A |
| FSL S5-523 | Thompson | |  | A |
| FSL R8-3597 | Tilene | |  | A |
| FSL R6-540 | Typhi | |  | A |
| FSL S5-536 | Typhimurium | |  | A |
| FSL S5-388 | Urbana | |  | B |
| FSL S5-410 | Urbana | |  | B |
| FSL S5-659 | Urbana | |  | B |
| FSL S5-660 | Urbana | |  | B |
| FSL S5-661 | Urbana | |  | B |
| FSL S5-961 | Virchow | |  | A |
| FSL R6-526 | Wandsworth | |  | A |
| FSL R8-1542 | Wangata | |  | A |
| FSL S5-438 | Weltevreden | |  | A |
| FSL S5-490 | Worthington | |  | A |

^a^ Isolates which were used as control in the population wide screen are marked with a †.

^b^ The genome sequence of S. enterica Typhimurium DT104 NCTC 13348 is available from http://www.sanger.ac.uk/resources/downloads/bacteria/salmonella.html.

^c^ A= Clade A, B= Clade B, X= mixed/ambiguous, n/a = not applicable.
