# Supplementary material for: Genome sequencing reveals diversification of virulence factor content and possible host adaptation in distinct subpopulations of Salmonella enterica
Source: BMC Genomics. 2011 Aug 22;12:425. doi: 10.1186/1471-2164-12-425 (PMC3176500; doi:10.1186/1471-2164-12-425)

Additional file 2. SNP-based maximum likelihood tree (A) and NeighborNet phylogenetic network (B) inferred from 8,779 SNPs found in the S. enterica subsp. enterica core genome. ML bootstrap values are based on 100 bootstrap replicates. The NeighborNet phylogenetic network was created with Splitstree4 v. 4.8 (Huson and Bryant, 2006, Mol Biol Evol 23:254-267). Bootstrap values <50% are not shown in the tree.

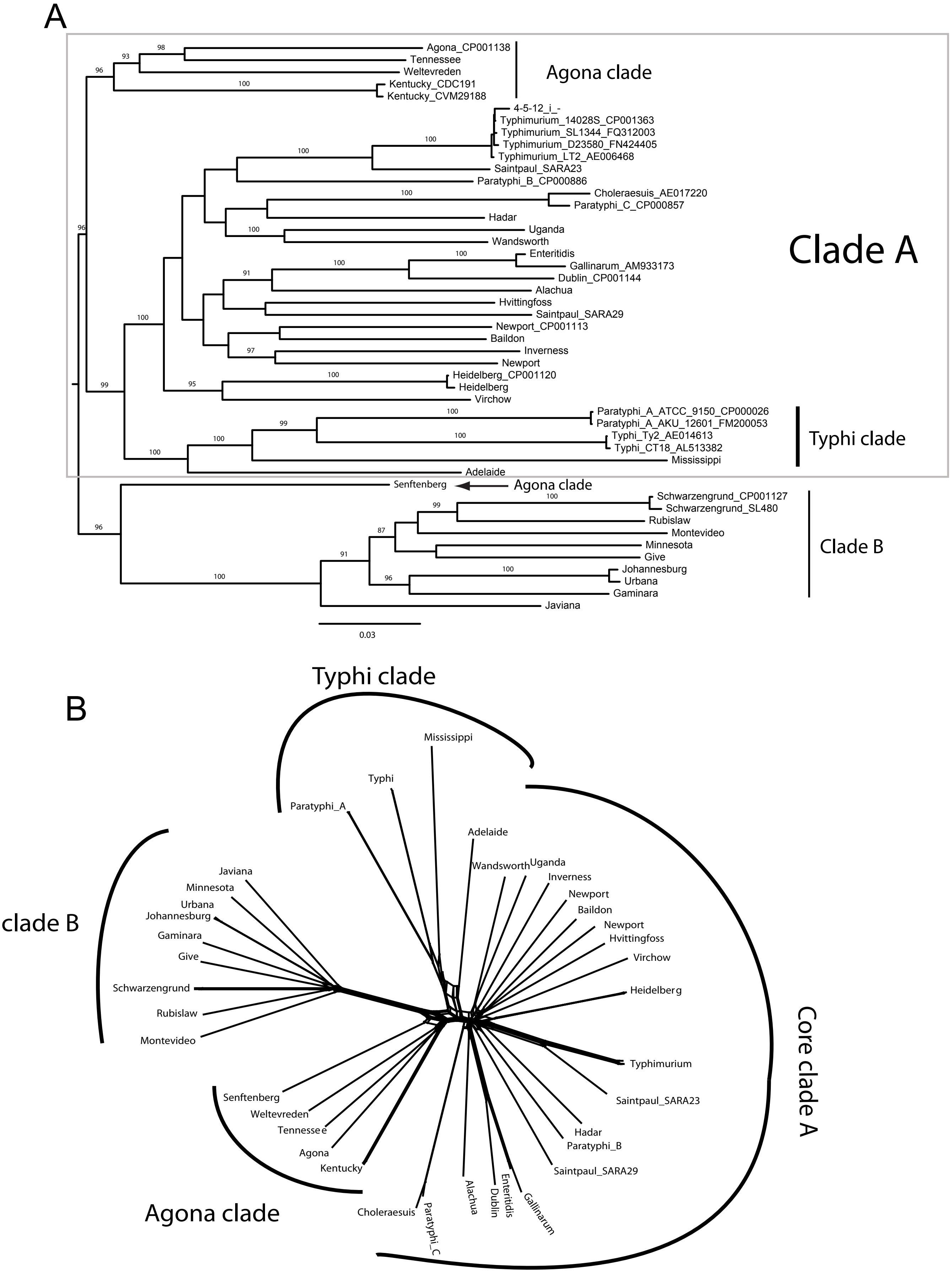

Supplement: Additional file 2 — SNP-based maximum likelihood tree and NeighborNet phylogenetic network inferred from 8,779 SNPs found in the S. enterica subsp. enterica core genome. PDF file containing results of phylogenetic analyses of core genome SNPs. [file 1471-2164-12-425-S2.PDF]
