# Supplementary material for: Genome sequencing reveals diversification of virulence factor content and possible host adaptation in distinct subpopulations of Salmonella enterica
Source: BMC Genomics. 2011 Aug 22;12:425. doi: 10.1186/1471-2164-12-425 (PMC3176500; doi:10.1186/1471-2164-12-425)

Additional file 3. Phylogenetic trees for individual genes and/or regions, without significant signal for recombination, located in the genomic islets SPI-18, CdtB-islet and the GICT18/1 (artAB) islet. Recombination/breakpoint analyses for SPI-18, CdtB-islet and GICT18/1 were performed using GARD (Kosakovsky Pond SL et al. 2006, Bioinformatics 22(24):3096-3098). Maximum likelihood phylogenies for the individual fragments between breakpoints were inferred with PAUP* 4.010b. Bootstrap values are based on 100 ML bootstrap replicates. bootstrap values <50% are not shown in the trees.

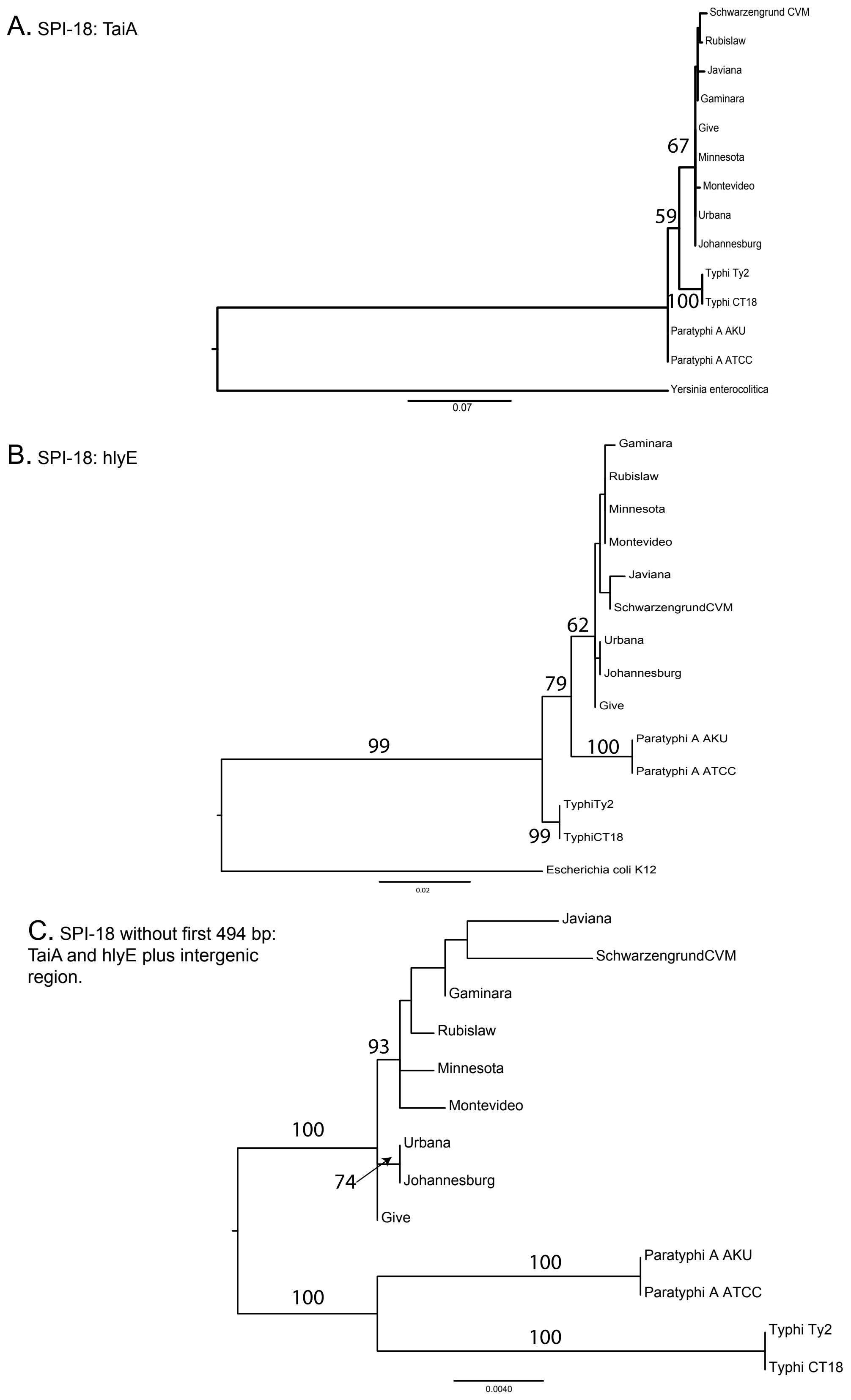
A. SPI-18: TaiA
Schwarzengrund CVM
Rubislaw
Javiana
Gaminara
Give
Minnesota
Montevideo
Urbana
Johannesburg
Typhi Ty2
Typhi CT18
Paratyphi A AKU
Paratyphi A ATCC
Yersinia enterocolitica
67
59
100
0.07
B. SPI-18: hlyE
Gaminara
Rubislaw
Minnesota
Montevideo
Javiana
SchwarzengrundCVM
Urbana
Johannesburg
Give
Paratyphi A AKU
Paratyphi A ATCC
TyphiTy2
TyphiCT18
Escherichia coli K12
62
79
99
100
99
0.02
C. SPI-18 without first 494 bp: TaiA and hlyE plus intergenic region.
Javiana
SchwarzengrundCVM
Gaminara
Rubislaw
Minnesota
Montevideo
Urbana
Johannesburg
Give
Paratyphi A AKU
Paratyphi A ATCC
Typhi Ty2
Typhi CT18
93
100
74
100
100
100
0.0040

D: CdtB-islet position1-543: IS related transposase (first 165 bp of fragment)

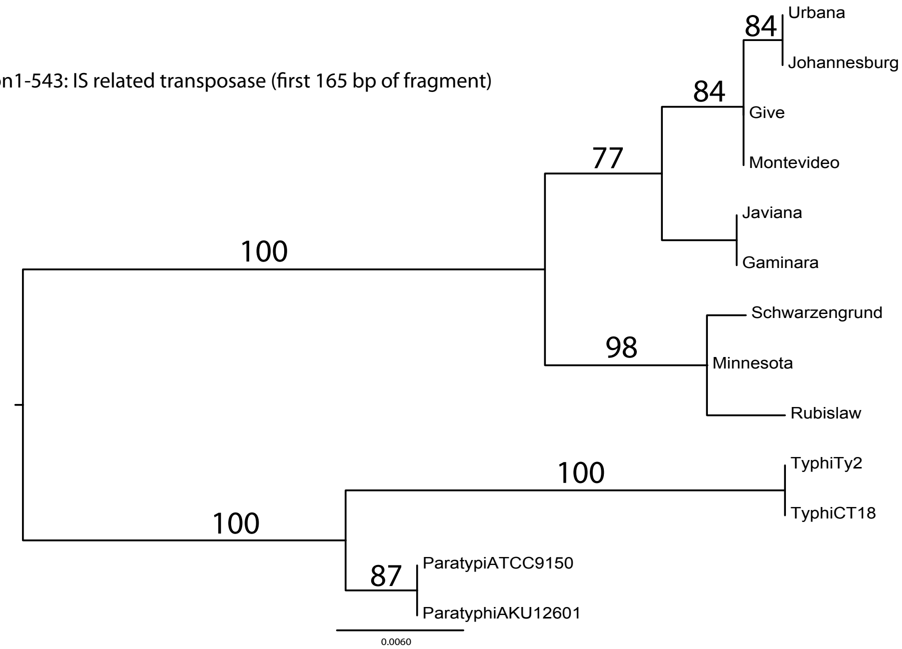

E. CdtB-islet position 544-2692: CtdB, prophage derived protein, hypothetical protein

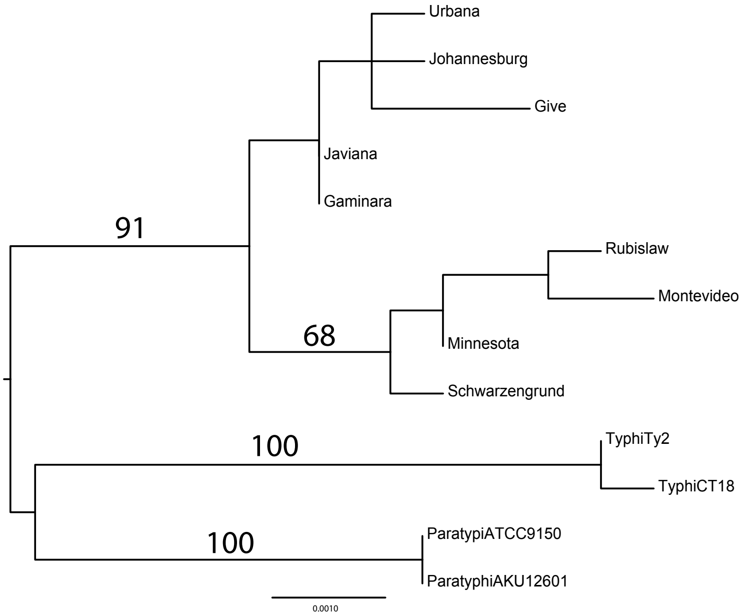

F. SPI-22 position 2693-3766 (mainly pltA part of pltB)

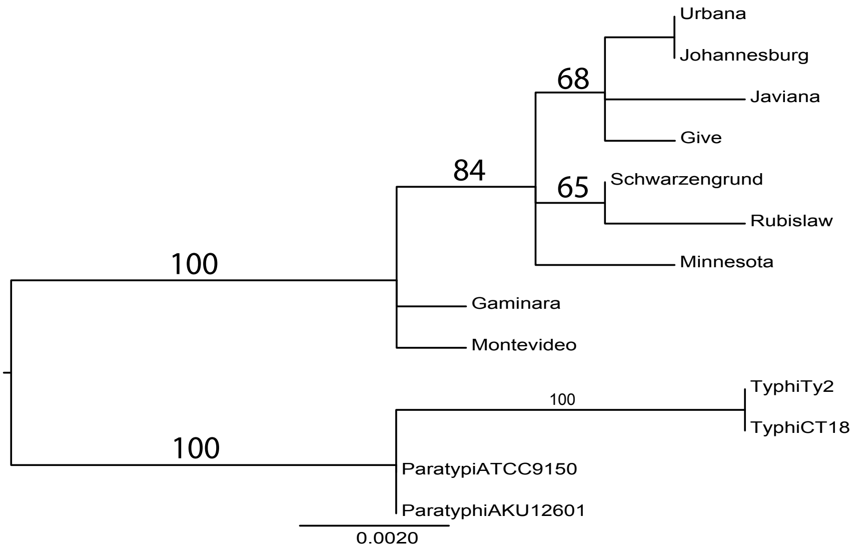

G: CdtB-islet position 3767-4495 pltB, small part of pltA

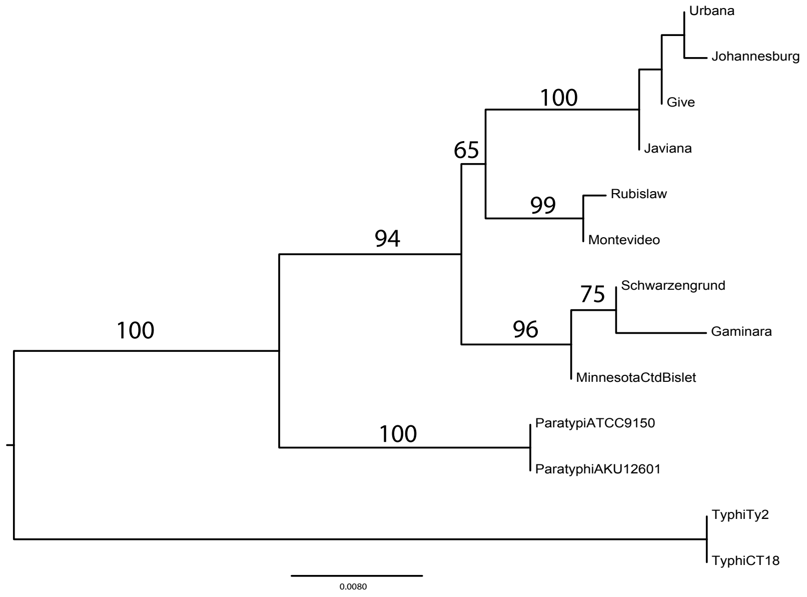

H: GICT18/1 insert: position1- 488 (integrase plus intergenic region)

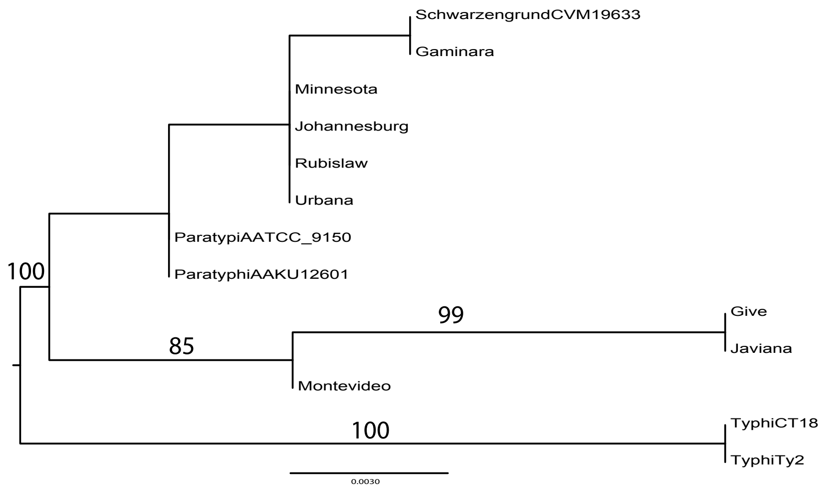

I: GICT18/1 insert, position 489 -4139.

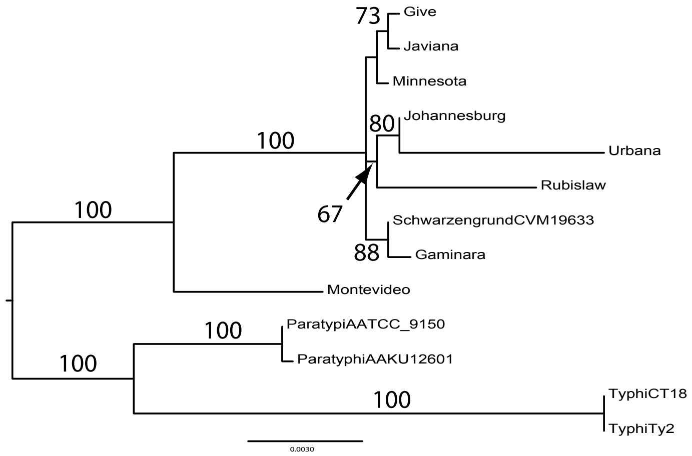

Supplement: Additional file 3 — Phylogenetic trees for individual genes and/or regions, without significant signal for recombination, located in SPI-18, the CdtB-islet and the GICT18/1 (artAB) islet. PDF file containing results of phylogenetic analyses of individual genes and/or regions, without significant signal for recombination, located in SPI-18, the CdtB-islet and the GICT18/1 (artAB) islet. [file 1471-2164-12-425-S3.PDF]
