# Supplementary material for: Genome sequencing reveals diversification of virulence factor content and possible host adaptation in distinct subpopulations of Salmonella enterica
Source: BMC Genomics. 2011 Aug 22;12:425. doi: 10.1186/1471-2164-12-425 (PMC3176500; doi:10.1186/1471-2164-12-425)
Supplement: Additional file 4 — Histogram of pairwise nucleotide divergence for selected combinations of Salmonella serovars. PDF file containing histograms of pairwise nucleotide divergence for selected combinations of Salmonella serovars. [file 1471-2164-12-425-S4.PDF]

Additional file 4. Histogram of pairwise nucleotide divergence for selected combinations of serovars. The nucleotide divergence of the top BLASTN hit for each ORF in the query genome was determined, and data were smoothed by arranging the ORFs in genome order and taking the running average with a window size of three ORFs. Completely conserved ORFs (= 0%) have been omitted from this figure. Comparison of *S.* Typhi and *S.* Paratyphi A (panel A) show a distinct bimodal distribution, a sharp peak of ca 0.0025 nucleotide divergence (arrow) and a diffuse peak of ca 0.01 nucleotide divergence. The low divergence peak has been explained as being the result of a large burst of (recent) homologous recombination between *S.* Typhi and *S.* Paratyphi A (Didelot et al, 2007, Genome Res 17:61-68). A similar comparison of clade A (*S.* Typhimurium) and clade B (serovars Schwarzengrund, Urbana, Rubislaw, Montevideo, Minnesota, Johannesburg, Give, Gaminara and Javiana) against *S.* Typhi does not show a bimodal distribution, indicating the absence of a large burst of homologous recombination between *S.* Typhi and these serovars.

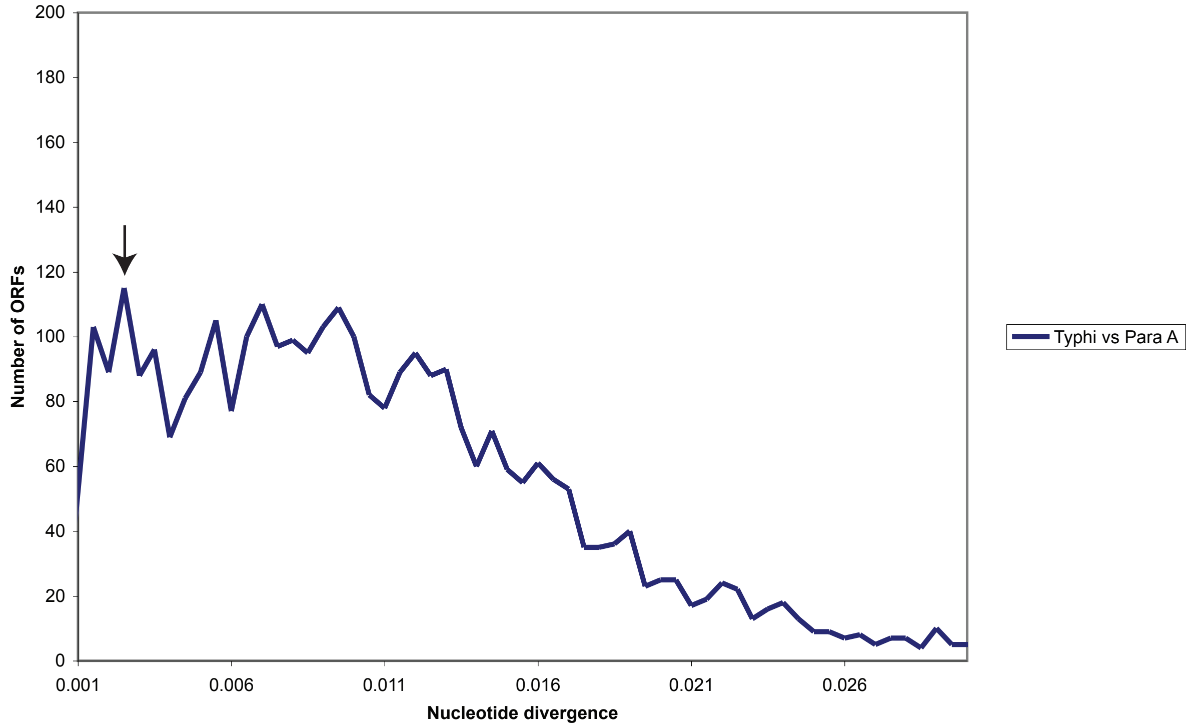
200
180
160
140
120
100
80
60
40
20
0
Number of ORFs
0.001
0.006
0.011
0.016
0.021
0.026
Nucleotide divergence
Typhi vs Para A

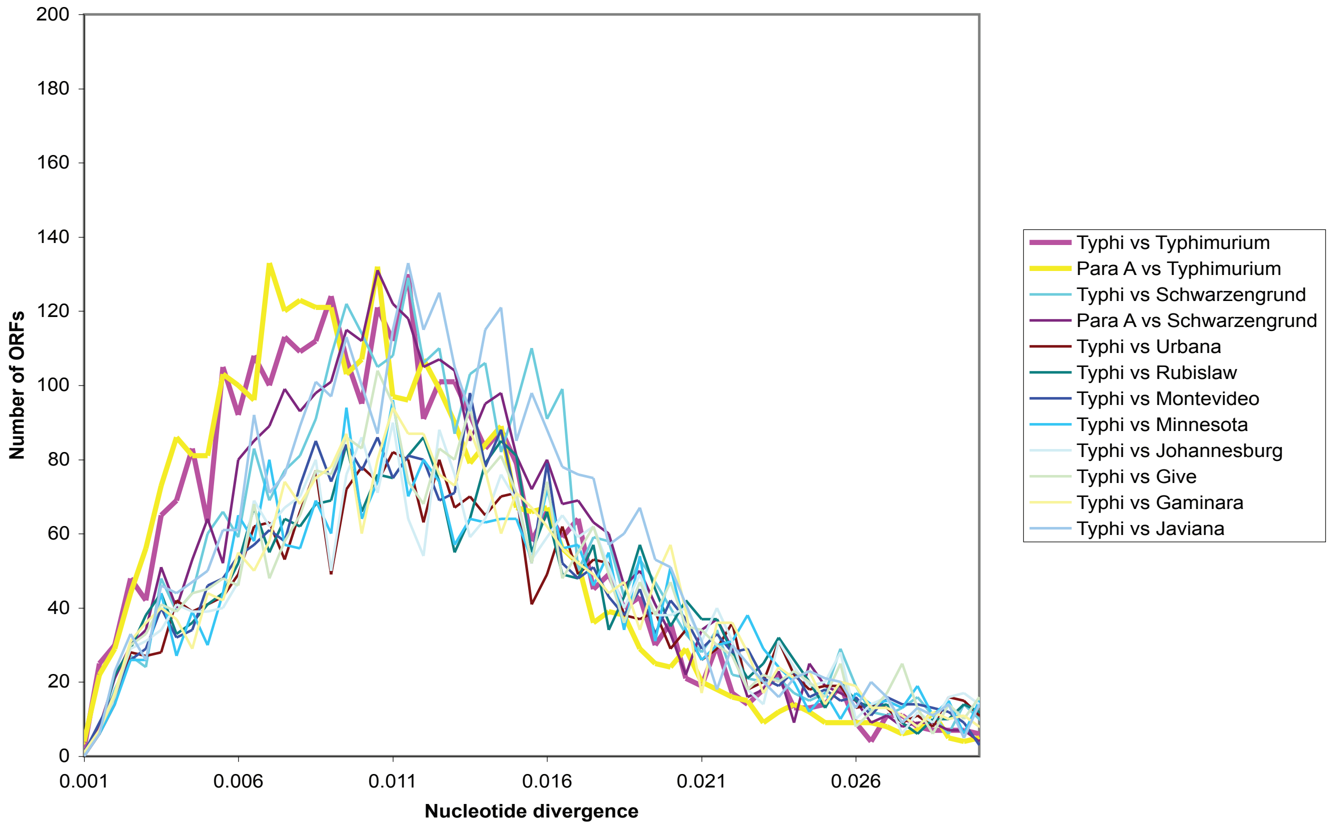
200
180
160
140
120
100
80
60
40
20
0
Number of ORFs
0.001
0.006
0.011
0.016
0.021
0.026
Nucleotide divergence
Typhi vs Typhimurium
Para A vs Typhimurium
Typhi vs Schwarzengrund
Para A vs Schwarzengrund
Typhi vs Urbana
Typhi vs Rubislaw
Typhi vs Montevideo
Typhi vs Minnesota
Typhi vs Johannesburg
Typhi vs Give
Typhi vs Gaminara
Typhi vs Javiana
