# Supplementary material for: Genome sequencing reveals diversification of virulence factor content and possible host adaptation in distinct subpopulations of Salmonella enterica
Source: BMC Genomics. 2011 Aug 22;12:425. doi: 10.1186/1471-2164-12-425 (PMC3176500; doi:10.1186/1471-2164-12-425)
Supplement: Additional file 5 — Draft genome assembly statistics for 16 S. enterica strains newly sequenced in this study. Word document containing an overview of the assembly statistics of the 16 newly sequenced Salmonella genomes. [file 1471-2164-12-425-S5.DOC]

**Additional file 5. Draft genome assembly statistics for 16 *S. enterica*** strains sequenced in this study.

| **Serovar** | **isolate** | **Genbank accession** | **Mean coverage** | **Number of contigs** | **Contig N50** | **Number of scaffolds*^a^*** | **Scaffold N50*^a^*** | **Genome size*^b^*** | **Number of ORFs*^c^*** | **RNA**  **Features^d^** |
| --- | --- | --- | --- | --- | --- | --- | --- | --- | --- | --- |
| Adelaide | FSL A4-669 | AFCI00000000 | 197 | 2,347 | 4,471 | 993 | 31,537 | 4,629,969 | 5,108 | 50 |
| Alachua | FSL R6-377 | AFCJ00000000 | 235 | 3,030 | 3,408 | 995 | 41,242 | 4,717,901 | 4,878 | 53 |
| Baildon | FSL R6-199 | AFCK00000000 | 156 | 2,003 | 5,526 | 727 | 77,666 | 4,713,356 | 4,981 | 59 |
| Gaminara | FSL A4-567 | AFCL00000000 | 228 | 2,272 | 4,672 | 873 | 39,817 | 4,636,159 | 5,027 | 57 |
| Give | FSL S5-487 | AFCM00000000 | 153 | 1,843 | 6,759 | 969 | 29,689 | 4,582,461 | 4,951 | 58 |
| Hvittingfoss | FSL A4-620 | AFCN00000000 | 187 | 2,049 | 5,210 | 798 | 46,086 | 4,706,818 | 5,002 | 58 |
| Inverness | FSL R8-3668 | AFCO00000000 | 213 | 2,414 | 4,797 | 1,029 | 29,892 | 4,983,034 | 5,487 | 54 |
| Johannesburg | FSL S5-703 | AFCP00000000 | 211 | 2,342 | 4,206 | 698 | 61,142 | 4,623,224 | 4,937 | 52 |
| Minnesota | FSL A4-603 | AFCQ00000000 | 268 | 2,696 | 3,472 | 724 | 68,635 | 4,563,323 | 4,986 | 55 |
| Mississippi | FSL A4-633 | AFCR00000000 | 143 | 2,032 | 5,606 | 750 | 71,013 | 4,783,829 | 5,045 | 54 |
| Montevideo | FSL S5-403 | AFCS00000000 | 181 | 1,780 | 7,978 | 896 | 53,282 | 5,072,621 | 5,275 | 53 |
| Rubislaw | FSL A4-653 | AFCT00000000 | 255 | 2,680 | 4,070 | 971 | 39,353 | 5,012,478 | 5,517 | 50 |
| Senftenberg | FSL A4-543 | AFCU00000000 | 168 | 2,558 | 4,607 | 1,006 | 51,735 | 4,972,530 | 5,388 | 60 |
| Uganda | FSL R8-3404 | AFCV00000000 | 178 | 1,873 | 7,564 | 1,157 | 25,556 | 4,774,305 | 5,139 | 56 |
| Urbana | FSL R8-2977 | AFCW00000000 | 220 | 2,784 | 3,805 | 1,059 | 28,403 | 4,832,852 | 5,435 | 60 |
| Wandsworth | FSL A4-580 | AFCX00000000 | 200 | 2,407 | 4,550 | 841 | 45,470 | 4,815,955 | 5,191 | 56 |

*^a^*After splitting scaffolds at putative discontinuities, identified by alignment to the S. Enteritidis and S. Typhimurium genomes (see Materials and Methods)

*^b^*Sum of scaffold lengths

*^c^*After merging ORFs that are split by gaps between contigs (see Materials and Methods)

^d^The number of RNA features (i.e., tRNAs and rRNAs) is low due to the fact that these are draft genomes. The expected number would be approximately 200 (215 in S. Typhimurium LT2).
