# Supplementary material for: Genome sequencing reveals diversification of virulence factor content and possible host adaptation in distinct subpopulations of Salmonella enterica
Source: BMC Genomics. 2011 Aug 22;12:425. doi: 10.1186/1471-2164-12-425 (PMC3176500; doi:10.1186/1471-2164-12-425)
Supplement: Additional file 8 — Primers and probes used for quantitave PCR experiments. Word document containing sequences of primers and probes used for quantitave PCR based population screen [file 1471-2164-12-425-S8.DOC]

Additional file 8. Primers and probes used for quantitave PCR based population screen

| **Assay ID** | **Type** | **Forward Primer** | **Reverse Primer** | **Probe 1 (FAM-MGB)^a^** | **Probe 2 (VIC-MGB)^a^** | **Clade A match** | **Clade B match** |
| --- | --- | --- | --- | --- | --- | --- | --- |
| 1089362 | SNP | CGGCGTGCGATCGATAC | GGTATGGGAACCACCACGAT | CAAAATGGCCGCcTCGC | ATCAAAATGGCCGCgTCGC | Probe 2 | Probe 1 |
| 3778429 | SNP | CGCGCCAGCGATATGG | GACGCCGTTGTTGTGGATTTA | ACGGAAATCaTCACGTTGACG | ACGGAAATCgTCACGTTGACG | Probe 2 | Probe 1 |
| 4244055 | SNP | TCTCCAAAATGCTGAACACTTGCTA | ACATCGTCTGGTCCGCAAAA | TTTCAGaCCCAGAATACG | TTTCAGgCCCAGAATACG | Probe 2 | Probe 1 |
| *hlyE* | Presence/Absence | AGTTCACAAAGTTTCAACAACGCTT | CCACCTGTGACTGGAAATAACTACTT | CTGCTGGCATTAGATAGC | - |  |  |
| *cdtB* | Presence/Absence | GTTTCCAGACAAAGAGCGGATAATG | GGCGAGATGCGACAGTTG | TCGGACGCAAGACATAAA | - |  |  |
| *pltA* | Presence/Absence | GCTCCCATCCATCACCTATCTG | ACATACTCTCTTTGCAATCGCATCA | TCGTTTTTTCATAAGCATTAAAG | - |  |  |
| *pltB* | Presence/Absence | ATAAAAACGGTTAAAGCTAACGGT | GCCCATATGCTCTGCTTTGAT | CCGCACATGCAACAAC | - |  |  |
| *artA* | Presence/Absence | CGCCGTCTGTTGCTTATATTGAGT | CAATTGGGATAGAATTTACAGATACA | CACTCGCTCAAAATGA | - |  |  |

^a^Actual SNP nucleotide is denoted by lower case letter.
